# Climate change and malaria: some recent trends of malaria incidence rates and average annual temperature in selected sub-Saharan African countries from 2000 to 2018

**DOI:** 10.1186/s12936-023-04682-4

**Published:** 2023-08-28

**Authors:** Walter Leal Filho, Julia May, Marta May, Gustavo J. Nagy

**Affiliations:** 1 Research and Transfer Centre “Sustainable Development and Climate Change Management”, Hamburg University of Applied Sciences, Ulmenliet 20, 21033 Hamburg, Germany; 2 Department of Natural Sciences, Manchester Metropolitan University, Manchester, M15 6BH UK; 3 Instituto de Ecología y Ciencias Ambientales, Facultad de Ciencias, Universidad de la República, UdelaR, Montevideo, Uruguay

**Keywords:** Malaria incidence rates, sub-Saharan countries, Public Health, Temperature, 2000–2018, Climate vulnerability

## Abstract

**Background:**

Malaria is still a disease of massive burden in Africa, also influenced by climate change. The fluctuations and trends of the temperature and precipitation are well-known determinant factors influencing the disease’s vectors and incidence rates. This study provides a concise account of malaria trends. It describes the association between average temperature and malaria incidence rates (IR) in nine sub-Saharan African countries: Nigeria, Ethiopia, South Africa, Kenya, Uganda, Ghana, Mozambique, Zambia and Zimbabwe. The incidence of malaria can vary both in areas where the disease is already present, and in regions where it is present in low numbers or absent. The increased vulnerability to the disease under increasing average temperatures and humidity is due to the new optimal level for vector breeding in areas where vector populations and transmission are low, and populations are sensitive due to low acquired immunity.

**Methods:**

A second source trend analysis was carried out of malaria cases and incidence rates (the number of new malaria cases per 1000 population at risk per year) with data from the World Health Organization (WHO) and average annual mean temperature from 2000 to 2018 from the World Bank’s Climate Change Knowledge Portal (CCKP). Additionally, descriptive epidemiological methods were used to describe the development and trends in the selected countries. Furthermore, MS Excel was chosen for data analysis and visualization.

**Results:**

Findings obtained from this article align with the recent literature, highlighting a declining trend (20–80%) of malaria IR (incidence rate) from 2000 to 2018. However, malaria IR varies considerably, with high values in Uganda, Mozambique, Nigeria and Zambia, moderate values in Ghana, Zimbabwe, and Kenya, and low values in South Africa and Ethiopia in 2018. Evidence suggests varying IRs after average temperature fluctuations in several countries (e.g., Zimbabwe, Ethiopia). Also, an inverse temperature-IR relationship occurs, the sharp decrease of IR during 2012–2014 and 2000–2003, respectively, occurred with increasing average temperatures in Ghana and Nigeria. The decreasing trends and fluctuations, partly accompanying the temperature, should result from the intervention programmes and rainfall variability. The vulnerability and changing climate could arrest the recent trends of falling IR.

**Conclusion:**

Thus, malaria is still a crucial public health issue in sub-Saharan Africa, although a robust decreasing IR occurred in most studied countries.

**Supplementary Information:**

The online version contains supplementary material available at 10.1186/s12936-023-04682-4.

## Background

Malaria is a disease that poses a significant burden to the health systems of many African countries. As the climate changes, shifts in temperature in geographic locations suitable for the transmission of the disease will occur, which will also require changes in the ways to handle it [1]. Malaria transmission is dependent on a combination of various climatic factors and human activity. Increases in greenhouse gas emissions are likely to exacerbate climate change in the future [2]. Furthermore, as more CO_2_ is released into the atmosphere, and temperature increases, a positive impact on vector breeding is seen, increasing the habitat index for reproduction. The main vectors of malaria transmission are mosquitoes [3], whose increase is likely to lead to more intensive malaria transmission [4], unless appropriate preventive measures are taken.

**Fig. 1:**
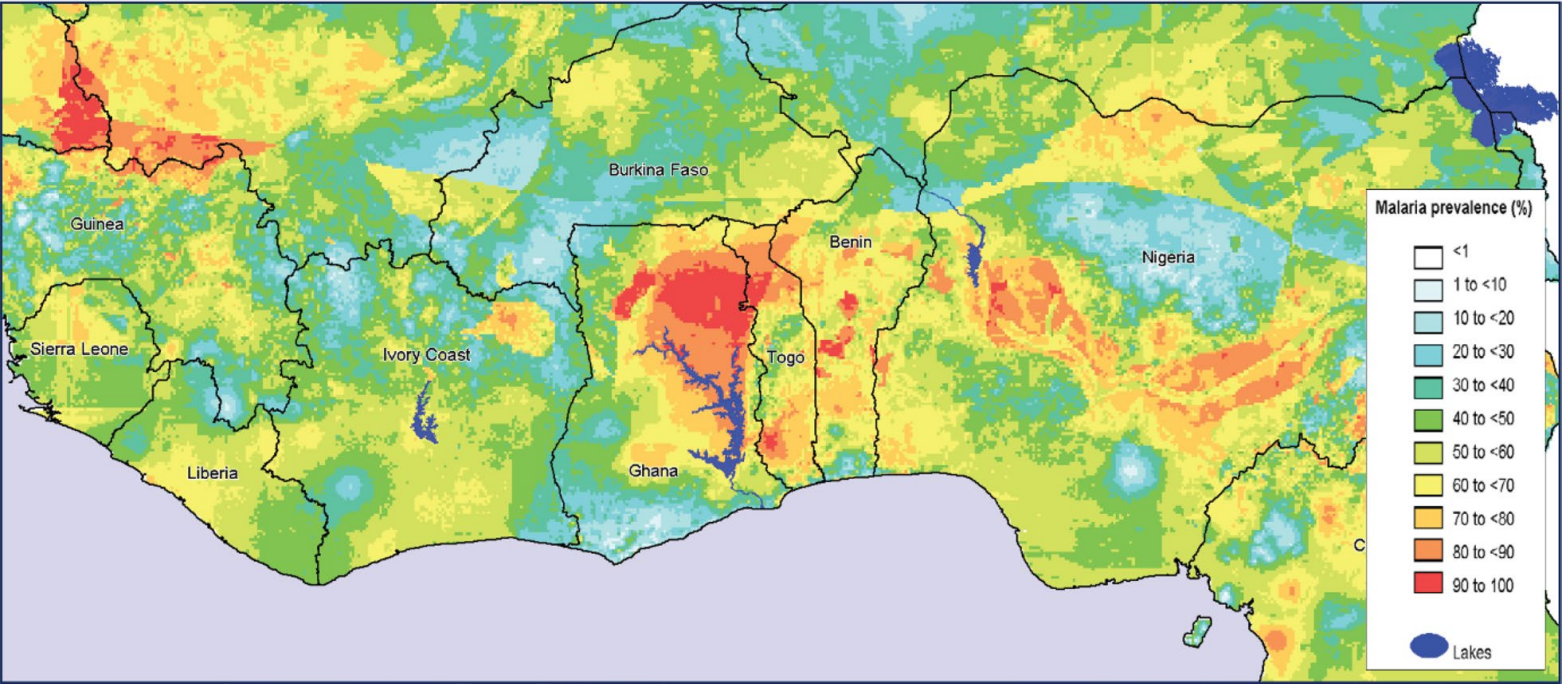
Occurrence and prevalence of malaria in West Africa (Source: Mapping Malaria Risk in Africa (MARA/ARMA) database)

Climate change further increases the risk of extreme temperatures occurring. Due to fluctuating temperatures, areas that generally experience low transmission rates may see differences attributable to increasing temperatures that rise to the optimal level for vector breeding. In addition, the transmission could be increased in susceptible regions due to the increased number of vectors and the low immunity of residents (e.g. in Ethiopia). The latter results from malaria not typically prevalent in each region [5]. Additionally, higher air humidity levels correlate to increased transmission of malaria. Finally, elevated humidity is associated with climate change, now occurring in regions where malaria is not as common but faces increased transmission risk [6]. Malaria is a significant public health problem in regions such as West Africa. According to the World Health Organization (WHO), the region bears a disproportionately high share of the global malaria burden. In 2017, an estimated 231 million malaria cases and 405,000 malaria deaths occurred in the region. Malaria is endemic to all countries in West Africa and is one of the region’s leading causes of death and disease [7]. Figure 1 presents a map where the occurrence of malaria in West Africa and the prevalence levels are outlined.

It is widely acknowledged that erratic rainfall patterns are expected to increase as climate change worsens. Therefore, areas that generally have drier conditions may experience heavy rainfall. An increase in moist environments provides optimum conditions for vector breeding and thus increases vector numbers in previously unexpected places. The latter results in an increased transmission rate, infection, and possible mortality due to the low immunity of the population [8]. However, heavy rainfall may also reduce the transmission rate by washing away mosquito vectors’ breeding sites.

Climate change is expected to cause an increase in the frequency of natural disasters (such as droughts or floods), usually leading to a disruption in access to essential resources such as clean drinking water, food, sanitation, as well as medical attention. In addition, such events promote the outbreak of various diseases, further exacerbated by floods, which increase moisture in the area and promote the reproduction of vectors through increased breeding sites and optimum conditions [9].

Since Africa is the world region most affected by malaria, there is a perceived need for studies that foster an understanding of how incidence levels correlate with temperature. On this basis, this paper departs from the research question, “To what extent does temperature influence malaria in sub-Saharan Africa”? This paper is structured as follows: Further to this background section, the next section describes the methods used, followed by a “Results and discussions” section, where the findings are presented and discussed. Finally, a “Conclusions” section, where the main lessons from the paper are drawn, and some prospects are outlined.

## Methods

**Fig. 2:**
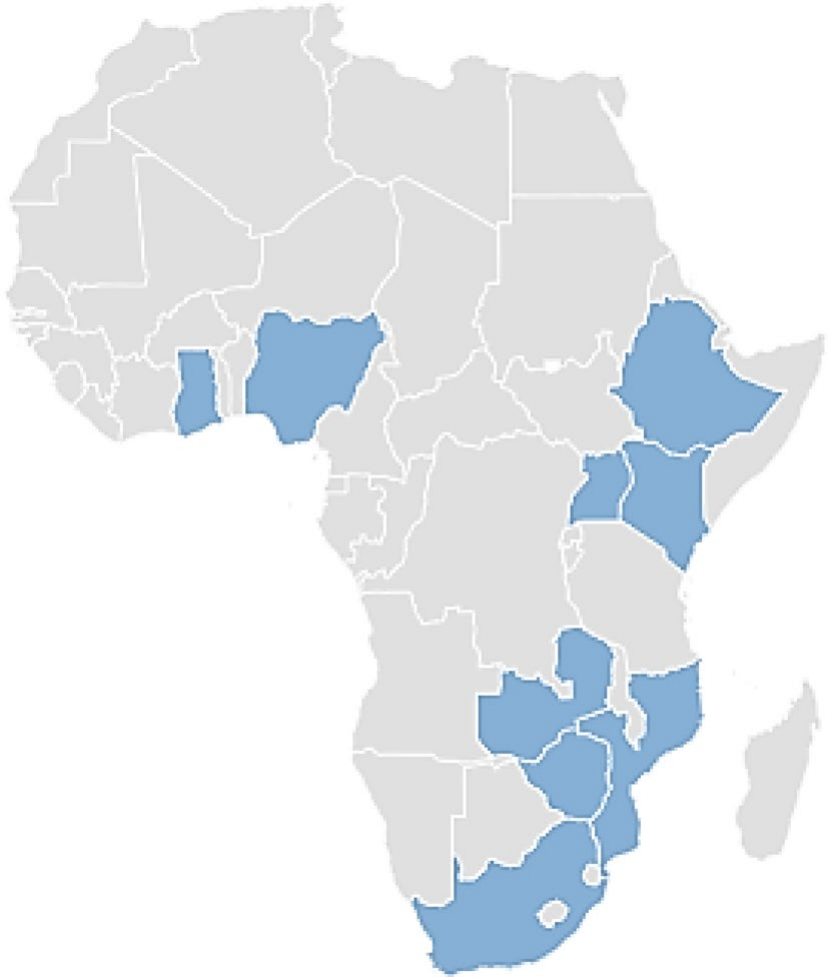
Illustrates the nine selected African countries in this study sample. Schematic map of Africa showing the nine studied countries: Nigeria, Ethiopia, South Africa, Kenya, Uganda, Ghana, Mozambique, Zambia, Zimbabwe

Against this background, this study examines the malaria situation based on the incidence rate in a sample of nine sub-Saharan African countries. These countries were selected due to the fact that sub-Saharan Africa accounts for the majority of global malaria infections [10]. For this purpose, this study provides a concise account of malaria trends and describes the association between temperature and malaria incidence rates in particular countries in the African context. These countries were chosen based on two main criteria: the interest to collect data from eastern, western and southern Africa, and the wish to include countries at different stages of development, including least developed countries (LDCs) such as Ethiopia, Mozambique and Zimbabwe, as well as more developed countries encompassing, for example, South Africa. Figure 2 illustrates the nine selected African countries in this study sample.

**Fig. 3:**
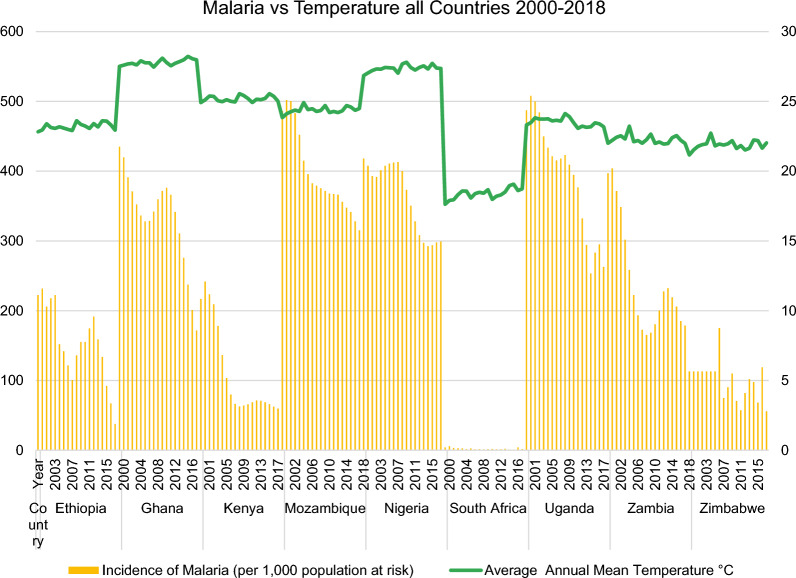
Incidence rates of malaria in nine sub-Saharan African countries from the World Health Organization (WHO) [11] and the average annual mean temperature from the World Bank’s Climate Change Knowledge Portal (CCKP) [13]

With regards to data collection, data on annual malaria incidence rates in the period 2000–2018 were obtained from the WHO [11] for the following selected sub-Saharan African countries: Ethiopia, Ghana, Kenya, Mozambique, Nigeria, South Africa, Uganda, Zambia and Zimbabwe (Fig. 3).

**Fig. 4:**
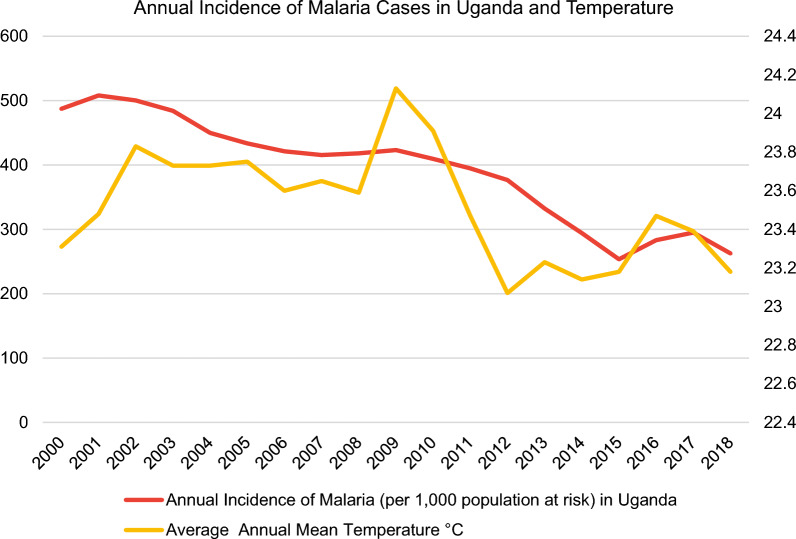
Annual incidence rates of malaria per 1000 population at risk and temperature in Uganda from the World Health Organization (WHO) [11] and the average annual mean temperature from World Bank’s Climate Change Knowledge Portal (CCKP) [13]

**Fig. 5:**
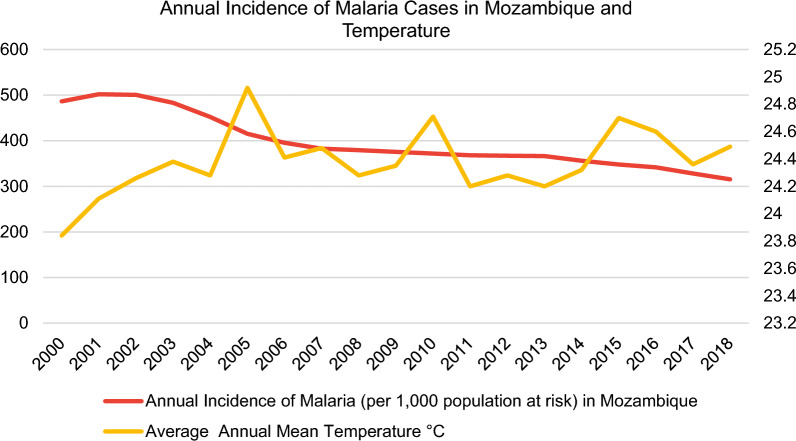
Annual incidence rates of malaria per 1000 population at risk in Mozambique from the World Health Organization (WHO) [11] and the average annual mean temperature from the World Bank’s Climate Change Knowledge Portal (CCKP) [13]

**Fig. 6:**
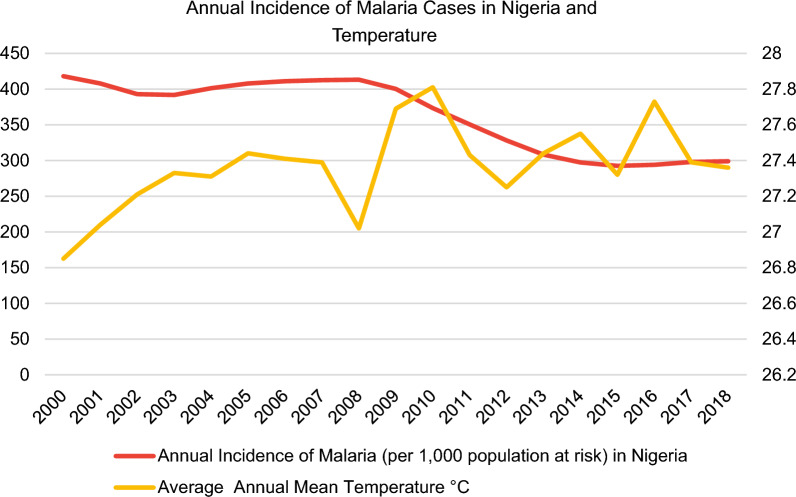
Annual incidence rates of malaria per 1000 population at risk and temperature in Nigeria from the World Health Organization (WHO) [11] and the average annual mean temperature from World Bank’s Climate Change Knowledge Portal (CCKP) [13]

**Fig. 7:**
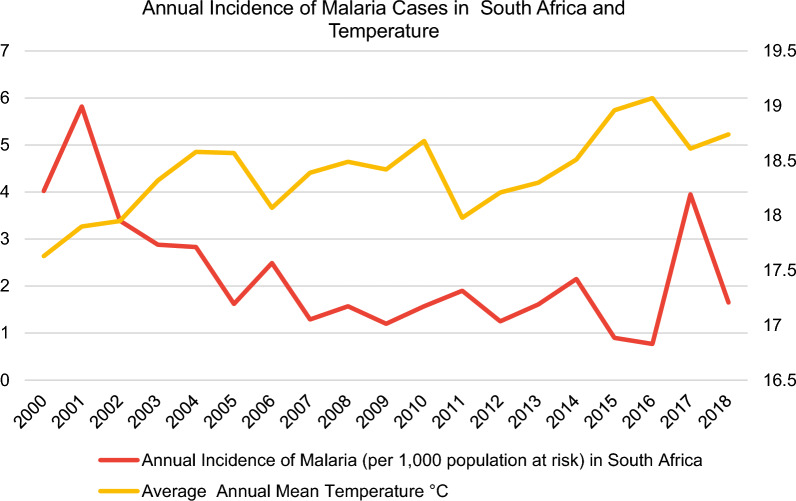
Annual incidence rates of malaria per 1000 population at risk and temperature in South Africa from the World Health Organization (WHO) [11] and the average annual mean temperature from the World Bank’s Climate Change Knowledge Portal (CCKP) [13]

**Fig. 8:**
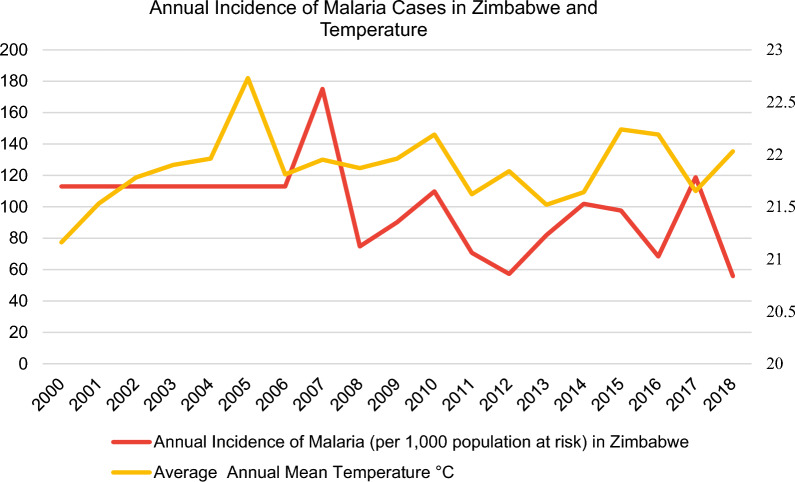
Annual incidence rates of malaria per 1000 population at risk in Zimbabwe from the World Health Organization (WHO) [11] and the average annual mean temperature from the World Bank’s Climate Change Knowledge Portal (CCKP) [13]

**Fig. 9:**
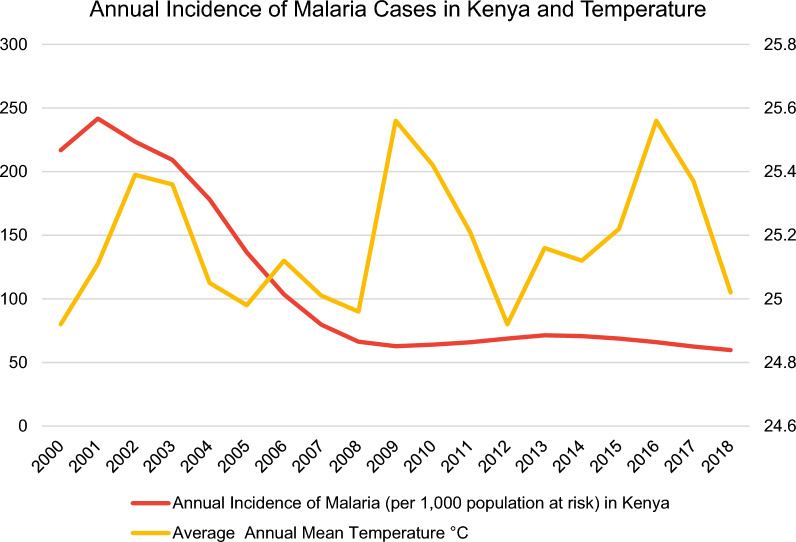
Annual incidence rates of malaria per 1000 population at risk and temperature in Kenya from the World Health Organization (WHO) [11] and the average annual mean temperature from World Bank’s Climate Change Knowledge Portal (CCKP) [13]

**Fig. 10:**
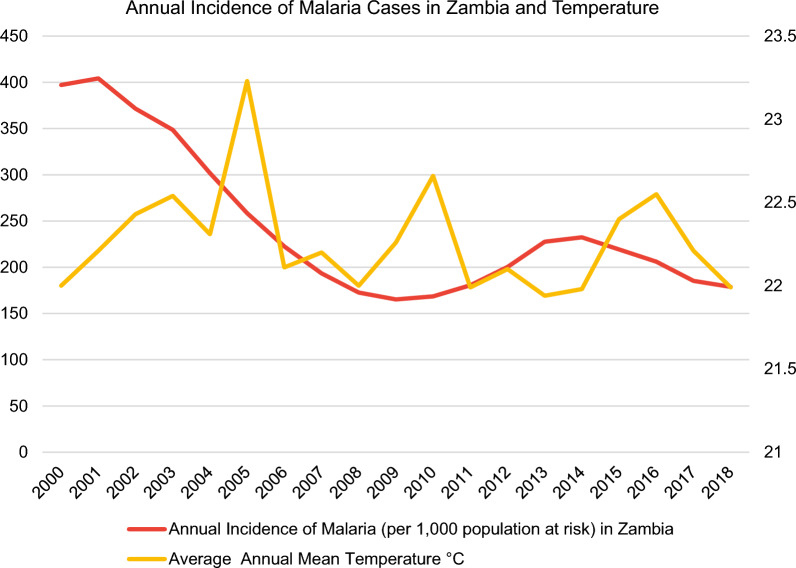
Annual incidence rates of malaria per 1000 population at risk and temperature in Zambia from the World Health Organization (WHO) [11] and the average annual mean temperature from World Bank’s Climate Change Knowledge Portal (CCKP) [13]

**Fig. 11:**
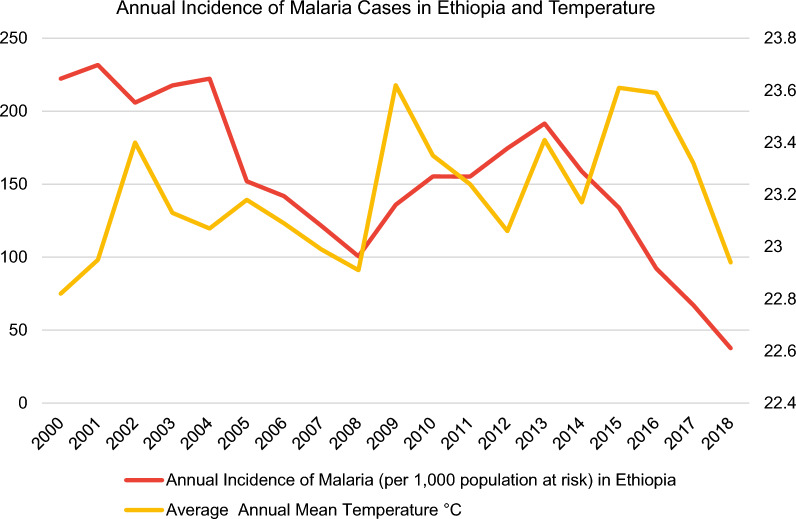
Annual incidence rates of malaria per 1000 population at risk and temperature in Ethiopia from the World Health Organization (WHO) [11] and the average annual mean temperature from the World Bank’s Climate Change Knowledge Portal (CCKP) [13]

**Fig. 12:**
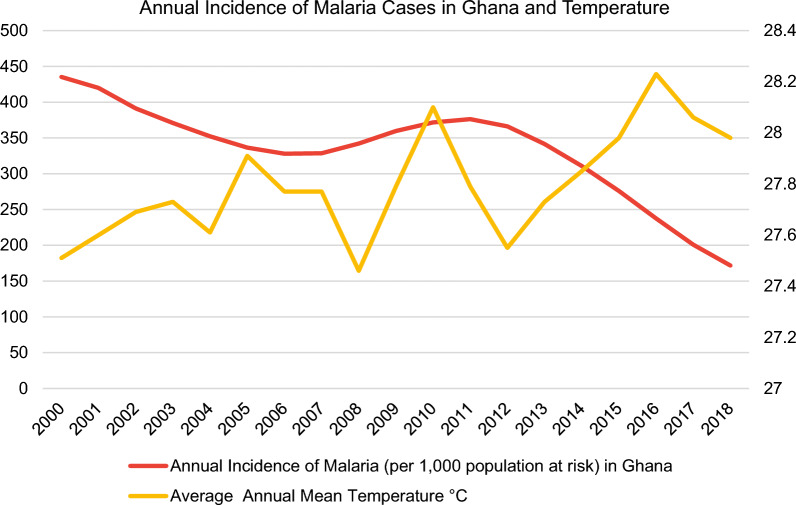
Annual incidence rates of malaria per 1000 population at risk and temperature in Ghana from the World Health Organization (WHO) [11] and the average annual mean temperature from World Bank’s Climate Change Knowledge Portal (CCKP) [13]

In this regard, the malaria incidence rate is defined as the number of malaria cases per 1000 population at risk yearly. By definition, the population at risk refers to those population inhabiting areas with malaria transmission [12]. Moreover, country-level datasets for the average annual mean temperature in °C (Fig. 3) were retrieved directly from the World Bank’s Climate Change Knowledge Portal (CCKP) [13] for the aforementioned sample of nine African countries in 2000–2018. The figures about the annual incidence rates of malaria per 1000 population at risk and average annual mean temperature (°C) of the nine selected countries (Figs. 3, 4, 5, 6, 7, 8, 9, 10, 11 and 12) were built with data from the WHO [11] and CCKP [13]. The average annual mean temperature for the respective sample countries are provided separately as Additional files 1, 2, 3, 4, 5, 6, 7, 8 and 9. Additional file 5 presents a complete overview of all selected countries with annual incidence of malaria and average annual mean temperature.

Additionally, descriptive epidemiological methods were used to elucidate the trend of malaria in the selected African countries. Furthermore, MS Excel was used for data analysis and visualization. For this reason, the left y-axis corresponds with the annual malaria incidence rate per 1000 population at risk, and the right y-axis corresponds with the average temperature in °C. At the same time, the x-axis encompasses the period 2000–2018.

## Results and discussion

According to the World Malaria Report (2020) released by the WHO [14], malaria constitutes a severe public health problem. Globally, roughly 229 million malaria cases were reported, and 409,000 deaths were attributed to malaria in 2019 [14]. In particular, Africa bears the most significant malaria burden as it accounts for 94% of all cases worldwide, with estimates of 215 million cases in 2019 [14].

Figure 3 illustrates the malaria incidence rate per 1000 population at risk in 2000–2018 for Ethiopia, Ghana, Kenya, Mozambique, Nigeria, South Africa, Uganda, Zambia and Zimbabwe. Additionally, the average annual mean temperature in °C is visualized and depicted.

Worldwide, malaria cases have drastically declined in recent years. According to the World Malaria Report [14], malaria case incidence reduced by 27% globally in the period 2000–2015, while according to Cibulskis et al. [15], mortality rates have dropped by 60% from 2000 to 2015.

Furthermore, the African region exhibited a considerable reduction in malaria incidence from 363 to 225 (38% reduction) cases per 1000 at-risk populations between 2000 and 2019 [14]. Additionally, malaria deaths decreased by roughly 44% in Africa from 680,000 to 386,000 in the period 2000–2019, while the malaria mortality rate declined by 67% between 2000 and 2019 from 121 to 40 deaths per 100 000 population at risk [14].

Based on estimates, malaria intervention programmes have substantially reduced malaria cases by 70% in sub-Saharan African countries [15]. It is essential to mention that insecticide-treated nets have hugely contributed to the decline of malaria cases in accordance with results by Bhatt et al. [16]. However, insecticide spraying is also used for preventing malaria transmission [17]; and anti-malarial drugs are utilized [18]. Notwithstanding, the African continent still bears a disproportionately heavy burden regarding malaria [18, 19]. In terms of symptoms of this vector-borne disease, it encompasses, for example, chills, fever, difficulty breathing, and headaches. Most importantly, malaria can lead to death, and children under 5 years are particularly vulnerable to this vector-borne disease accounting for 80% of all deaths [18].

In many respects, the results of the current literature are congruent with the results obtained in this article. Thus, Figs. 3, 4, 5, 6, 7, 8, 9, 10, 11 and 12 clearly illustrate an overall declining trend of malaria incidence rates observed across all selected African countries from 2000 to 2018. Therefore, this study substantiates preceding research as the trend of falling malaria incidence rates is consistent with those previously published by Cibulskis et al. in 2016 [15].

However, malaria incidence rates vary considerably among the sub-Saharan African countries. For example, comparing the rates for each selected country, Uganda reveals the highest incidence rate of malaria with 508 per 1000 population at risk in 2001, followed by Mozambique with 501.9 in 2001 and Nigeria with 418 in 2000, respectively, as demonstrated by Figs. 4, 5 and 6. Besides, these results align with some prior research findings in the World Malaria Report [14] published in 2020, identifying that the following three sub-Saharan African countries, Nigeria (27%), Uganda (5%) and Mozambique (4%), accounted for approximately 36% of all cases worldwide [14].

Regarding the malaria data for 2018, it is critical to note that in this study, Mozambique can be classified as the country with the highest malaria incidence rate of 315.3 per 1000 population at risk, followed by Nigeria with 299 and Uganda with 262.7. respectively.

According to the World Malaria Report, Nigeria, the most populous country in Africa, accounted for about 27% of malaria cases globally [14]. Consequently, this accentuates the fact that Nigeria is the country bearing the most significant malaria burden worldwide [20]. Based on estimates, 61 million cases were reported in 2019 [14]. Approximately 201 million of Nigeria’s population are exposed to the risk of malaria infection and therefore classified as the population at risk in 2019 [14]. However, after 8 years of stagnating malaria incidence rates between 2000 and 2008 at around 406 cases, Nigeria, a country in West Africa, experienced considerable declining incidence rates from 413.1 to 2008 to 299 in 2018 (27.6% reduction of IR), as depicted in Fig. 6.

As reported in the National Malaria Control Programme of the Republic of Mozambique, malaria poses a serious public health threat [21]. Apart from Nigeria, Mozambique, positioned in Southeastern Africa, suffers from one of the highest malaria burdens [14]. Besides, Zacarias and Andersson [22] conducted a study investigating the effects of temperature on malaria incidence where a maximum temperature exceeding 28 °C increased malaria risk. However, Mozambique’s malaria incidence rates dropped gradually from 501.9 to 2001 to 382.6 in 2007 (a reduction of IR by 23.77%). This period is followed by incidence rates remaining reasonably constant at roughly 370 cases per 1000 at-risk populations till 2013. Contrastingly, since 2013, the rates began to decline consistently to 315.3 in 2018, as depicted in Fig. 5 for Mozambique. The dominant modes of malaria variability in Mozambique were closely related to precipitation from 2010 to 2017, linked with the El Niño-Southern Oscillation. La Niña leads to wetter conditions over southern Mozambique, therefore, higher malaria prevalence [23].

By contrast, it is crucial to note that South Africa recorded the lowest malaria incidence rate of 0.77 per 1000 population at risk in 2016, with declining rates from 5.82 to 0.77 per 1000 population at risk during the observation period 2001–2016. Nevertheless, the malaria incidence rate increased to 3.95 per 1000 population at risk in South Africa in 2017, as evident in Fig. 7. This development was preceded by a rise in average temperature from 2011 to 2016. Regarding the incidence rates in 2018, South Africa can be categorized as having the lowest malaria incidence rate compared to other sub-Saharan African countries. Thus, it has become increasingly evident that South Africa demonstrates considerable improvements that have been made in tackling the malaria burden [24]. As exemplified by a study conducted by Moonasar et al. [25], a nearly 90% decrease in malaria cases was achieved from 63,663 to 6741 over the past decade from 2000 to 2010. These previous findings confirming such a substantial decline are congruent with results in Fig. 7 for South Africa (decline of IR by 60% from 2000 to 2010).

Besides South Africa, Zimbabwe demonstrates lower incidence rates with 55.83 per 1000 population at risk in 2018, as illustrated by Fig. 8, followed by Kenya with 59.75 in 2018, as depicted in Fig. 9 out of the nine selected sub-Saharan African countries.

Figure 9 clearly illustrates a steadily declining trend in Kenya, situated on the east coast of Africa. During the observation period from 2000 to 2009, the rate decreased markedly from 216.8 to 62.77 (71% reduction of IR), while remaining generally stable after 2009 with approximately 65 cases per 1000 population at risk.

However, malaria incidence rates differ in intensity and regularity between the countries. Uganda (from 2000 to 2018) and Zimbabwe (from 2006 to 2018) showed a fluctuating but decreasing incidence trend, as exemplified by Figs. 4 and 8. For example, in Uganda, situated in eastern Africa, fluctuations in incidence rates coincide with changes in the average annual mean temperature. As a result, higher malaria incidence rates can be observed with increasing temperature, evident in Fig. 4. For instance, the peak average annual mean temperature of 23.47 °C in 2016 preceded the peak incidence rate of 294.9 per 1000 population at risk in 2017. Conversely, a slight decrease in average temperature from 24.13 °C to 23.07 °C over 3 years (2009–2012) was accompanied by a considerable reduction of malaria incidence rates by around 100 per 1000 population at risk from 423.1 to 376.6 (11% reduction).

Considering the findings presented by Kigozi et al. [26], annual malaria incidence rates declined rapidly from 281.7 to 2016 to 170.0 cases per 1000 population in Uganda in 2018. However, malaria incidence rates in this article indicate a slower declining trend of 7.2%, decreasing from 283.1 cases in 2016 to 262.7 in 2018 per 1000 population. Besides, agreeing with previous results from the World Malaria Report in 2019 [10], Uganda was categorized as one of the greatest contributors to malaria cases at the global level in 2018 [10, 26].

As clearly indicated by study results in this article, fluctuating malaria incidence rates can also be observed in Zimbabwe from 2006, as visualized in Fig. 8. Before, from 2000 to 2006, the incidence rate remained stagnant at 113 cases per 1000 population. Additionally, the increasing trend of malaria occurred with increasing average temperature in Zimbabwe, located in Southeast Africa, from 2008 to 2010 and 2013 to 2014.

According to Fig. 8, rates rose from 74.73 to 109.8 per 1000 at-risk population in 2008–2010. At the same time, the average annual mean temperature slightly increased from 21.87 to 22.19 °C from 2008 to 2010. Moreover, it is imperative to note that a significant increase in temperature from 2004 to 2005 was accompanied by an increase in malaria cases in the following years 2005/2006 for Zimbabwe. Prior to this, a growing temperature trend from 2000 to 2004 did not lead to higher incidence rates. However, falling incidence rates from 2017 to 2018 were associated with rising average temperatures.

Indeed, Sande et al. [27] confirmed the overall trend of falling malaria incidence rates in Zimbabwe, identifying an 81.3% decline in malaria incidence from 155 to 29 cases per 1000 population in the period 2003–2015. Additionally, results obtained in this article elucidate low incidence rates for Zimbabwe, with 57.22 cases per 1000 population in 2012, as displayed in Fig. 8. This result is similar compared with the study mentioned above undertaken by Sande et al. [27], demonstrating a significant decline in malaria incidence rates for Zimbabwe to 22 per 1000 population in 2012. However, as visualized in Fig. 8, the cases per 1000 population have risen substantially for 2 consecutive years, from 57.22 to 2012 to 101.9 cases per 1000 population in 2014, before decreasing again to 97.61 in 2015. Subsequently, this increase complies with the results from the abovementioned study by Sande et al. [27], concluding an increase in malaria incidence rates in Zimbabwe since 2012.

In Zambia, there is a sharp IR decrease of about 60% (from 2001 to 2009), with an increased period of about 40% from 2009 to 2014. This decline is accompanied by highly fluctuating average temperatures and occurred despite rising average temperatures from 2001 to 2003, 2004 to 2005 and 2006 to 2007. Since 2005 the average temperature has decreased by − 1.2 °C. Notwithstanding, there is no clear overall average temperature trend from 2000 to 2018.

Nevertheless, according to a study carried out by Lubinda et al. [28], a gradual increase in malaria incidence can be observed in Zambia from 2008 to 2010. This particular finding is consistent with the results in this article. Correspondingly, Fig. 10 reflects a post-2008 trend of rising incidence rates by about 35%, from 172.6 to 2008 to 232.2 cases per 1000 population in Zambia in 2014. After 2014, IR started to decline again to 178.8 till 2018 but did not reach the lowest value from 2009.

In Ethiopia, located in East Africa, a robust decreasing IR trend of about 55% was observed from 2004 to 2008 and of about 80.4% from 2013 to 2018. Yet, from 2008 to 2013, IR rose and peaked in 2013. Therefore, the decreasing IR trend from 2005 to 2008, 2013 to 2014, and 2016 to 2018 coincides with decreasing average temperatures in these time periods. Also, an increase in average temperature from 2000 to 2001, 2008 to 2009 and 2012 to 2013 is associated with rising IR. By contrast, a reduction in IR from 2001 to 2002, 2004 to 2005, and 2014 to 2016 coincides with warming in these time spans.

The latest research findings by Taffese et al. [29], examining malaria epidemiology in Ethiopia, identified two peaks in malaria case incidence rates in 2004 and 2010. Moreover, these culminations correspond with study results in this article as particularly exemplified for 2004. In addition to peaks in IR in 2004 and 2010, peaks occurred in 2001 and 2013 (Fig. 11). Besides, the overall decreasing trend in malaria incidence rates by 84% from 231.7 to 37.61 for Ethiopia in 2001–2018 is manifested in Fig. 11 despite some annual variations as exemplified by an increase of about 8% occurring from 2002 to 2004 and by around 90% from 2008 to 2013.

Consequently, it is worth emphasizing that the main findings comply with the results presented in the study above by Taffese et al. [29], where a decline in incidence rates was observed between 2001 and 2016 despite fluctuations between 2002 and 2004 and 2008 and 2010. Furthermore, study results obtained in this article reveal that the downward trend in Ethiopian malaria incidence rates was pronounced from 2013 to 2018. Subsequently, this finding corresponds with the study by Taffese et al. [29], where malaria incidence rates exhibited a similar declining trend from 2013 to 2016.

Finally, malaria poses a serious public health challenge in Ghana due to its severe transmission [30]. Nevertheless, the Global Fund Report (2016) [31] highlights a noticeable decline in incidence by 45% for Ghana, a country in West Africa, in the time period 2000–2015. Notably, in this study, the decline aligns with the trend of falling incidence rates by about 60% from 435.2 to 171.7 in the period 2000–2018 despite a slight increase of 14.4% from 2007 to 2011, as evident in Fig. 12. Besides, there is no clear association between IR and average temperature despite some exceptions where falling IR coincides with declining average temperature, as exemplified in the periods from 2003 to 2004 and from 2016 to 2018. On the other hand, rising IR is observed with increasing average temperature (e.g., from 2008 to 2010). Further, a reduction in malaria cases occurs with an increase in average temperature from 2000 to 2003 and 2014–2016.

It is noteworthy that in particular countries temperature increases with rising IR over specifically selected periods. At the same time, temperature increases are accompanied by a decrease in IR in the respective countries. In contrast, temperature declines coincide in respective countries with IR increases or IR decreases. To sum up, a directly proportional relationship between temperature and IR and an inversely proportional relationship can be observed in selected countries. One explanation is that temperature is not the single determining factor influencing malaria transmission and, thus, incidence rates. Therefore, other factors, such as humidity, precipitation and geographical factors, are also relevant [32]. For this reason, these factors must be taken into consideration when analyzing incidence rates. However, some studies exclusively focus on the impact of temperature on malaria in Africa [1, 33–36].

Moreover, research performed by Thomson et al. [37] evaluated whether the impact of scaling up malaria control interventions in ten African countries on all-cause under-five mortality may overestimate or underestimate the impact of interventions or if there was no significant difference in climate suitability for malaria in the pre-and post-intervention period. The evaluation framework assessed whether the deployed interventions have impacted malaria morbidity and mortality and required consideration of climate-related transmission factors, e.g., drought, floods or higher temperatures. From the studied countries by these authors, three coincide with this study and lie in the three categories, Uganda (overestimated), Ethiopia (underestimated), and Mozambique (neutral), respectively.

### Summary of general risks and vulnerability from the literature

Due to this high number of malaria cases in sub-Saharan Africa, this study focused on nine sub-Saharan African countries from different regions and varying IR, including the most populated, namely Nigeria, Ethiopia and South Africa, as well as Kenya, Uganda, Ghana, Mozambique, Zambia and Zimbabwe.

Under the plausible mid-term futures, the expected warming and humidity changes will increase mosquito breeding by increasing the habitat index for reproduction and, therefore, malaria transmission. Besides the likely increases in extreme and fluctuating temperatures, the optimal level for vector breeding would be reached in areas where the number of vectors and disease transmission are low or not observed yet, where the populations are more sensitive (more vulnerable) due to low acquired immunity.

Additionally, rising humidity levels and rainfall associated with malaria transmission will also expand the disease. Malaria transmission in new areas is observed due to increased rainfall and humidity in previously dry regions. Also, vectors will likely wash out of their previous breeding sites and move to other locations.

These current and expected increased climate risk factors could partially arrest the recent declining world trends of malaria case incidence and mortality rates that decreased before by 27% [14] and 60% [15], respectively. The African region followed the world trend, reducing the number of deaths and mortality rates in Africa by 44% and 67%, respectively [14]. For instance, malaria intervention programmes decreased malaria cases by 70% in sub-Saharan African countries [15]. However, the African continent still bears a disproportionately heavy burden regarding malaria, accounting for 94% of all cases worldwide in 2019 [14].

Finally, the sub-Saharan African countries Nigeria (with a population of about 213 million people [38]) (27%), Uganda (5%) and Mozambique (4%) accounted for approximately 36% of all cases globally [14]. Though, this study did not consider all countries in sub-Saharan Africa where malaria occurs.

## Conclusions

The results from the literature align with the findings obtained in this article, highlighting an overall declining trend of malaria incidence rates despite fluctuations observed across the nine selected sub-Saharan countries from 2000 to 2018.

However, malaria incidence rates vary considerably between the studied countries, reaching high rates (cases per 1000 population at risk) in Uganda (508) and Mozambique (501.9) in 2001, followed by Nigeria (418) in 2000. Beyond interannual variability, the three countries mentioned above show the most cases. Despite a significant decrease, Mozambique showed the highest incidence rate (315.3) in 2018, followed by Nigeria with 299 in 2018 and Uganda with 262.7 in 2018.

The lowest incidence rate (IR) of the population at risk per 1000 occurred in South Africa, with a decrease from 5.82 to 0.77 in 2001–2016 despite an increase to 1.65 in 2018, remaining still the lowest IR in the region. Moreover, the following countries with low IR in 2018 are Kenya at 37.61, Zimbabwe at 55.83 and Kenya at 59.75.

The study has some limitations. The first one is that the data refers to trends associated with temperature and does not emphasize other variables, such as humidity or a combination of both. A further limitation is related to the fact that the trends investigated are limited to a sample of nine sub-Saharan African countries and need to be more significant to allow definitive conclusions to be drawn related to the African continent as a whole. The study nonetheless provides a welcome contribution to the literature since it has analyzed and documented trends related to malaria in some of the countries in Africa most affected by it and which are seldom investigated in a combined way. In addition, the geographical distribution of the sample also offers a rough profile of how malaria is associated with climate change in the region, hence helping to foster a broader understanding of the international implications of this important topic.

The contribution of the paper to the literature can be better understood by looking at some trends it has identified, such as:

(i) Malaria continues to be a disease of significant proportions in Africa. However, the findings obtained in this article align with the literature mentioning changes in incidence rates observed across the nine selected sub-Saharan countries in the period 2000–2018.
(ii) Mozambique, Nigeria and Uganda show the highest incidence rates among the studied countries, while South Africa, Zimbabwe and Kenya show the lowest incidence rates among the studied countries.
(iii) The average annual mean temperature fluctuating trends seem to precede the incidence rate variations by 1 year, like in Zimbabwe. Also, from 2005 to 2006, the decrease in the incidence rate in Zambia accompanies the decrease in temperature since 2005. Further, the sharp decrease in the incidence rate in Ethiopia from 2004 to 2016 coincides with a warming period from 2008 to 2016, which is probably beyond the temperature threshold acceptable for mosquitoes to breed.
(iv) In the future, it is advisable to pay better attention to temperature trends in initiatives focusing on approaches to malaria control in the region, including vector control, early diagnosis and treatment, and community-based interventions.

### Supplementary Information

**Additional file 1.** Average annual mean temperature for Ethiopia downloaded from Climate Change Knowledge Portal (Available at: https://climateknowledgeportal.worldbank.org/country/ethiopia/climate-data-historical).

**Additional file 2.** Average annual mean temperature for Ghana downloaded from Climate Change Knowledge Portal (CCKP) (Available at: https://climateknowledgeportal.worldbank.org/country/ghana/climate-data-historical).

**Additional file 3.** Average annual mean temperature for Kenya downloaded from Climate Change Knowledge Portal (CCKP) (Available at: https://climateknowledgeportal.worldbank.org/country/kenya/climate-data-historical).

**Additional file 4. ** Average annual mean temperature for Mozambique downloaded from Climate Change Knowledge Portal (CCKP) (Available at: https://climateknowledgeportal.worldbank.org/country/mozambique/climate-data-historical).

**Additional file 5.** Annual incidence of malaria (per 1,000 population at risk) with data from World Health Organization (WHO) (available at: https://www.who.int/data/gho/data/indicators/indicator-details/GHO/malaria-incidence-(per-1-000-population-at-risk) and average annual mean temperature with data from Climate Change Knowledge Portal (CCKP) (available at: https://climateknowledgeportal.worldbank.org/) for selected countries in study sample: Nigeria, Ethiopia, South Africa, Kenya, Uganda, Ghana, Mozambique, Zambia and Zimbabwe.

**Additional file 6.** Average annual mean temperature for Nigeria downloaded from Climate Change Knowledge Portal (CCKP) (Available at: https://climateknowledgeportal.worldbank.org/country/nigeria/climate-data-historical).

**Additional file 7. **Average annual mean temperature for Uganda downloaded from Climate Change Knowledge Portal (CCKP) (Available at: https://climateknowledgeportal.worldbank.org/country/uganda/climate-data-historical). 

**Additional file 8. **Average annual mean temperature for Zambia downloaded from Climate Change Knowledge Portal (CCKP) (Available at: https://climateknowledgeportal.worldbank.org/country/zambia).

**Additional file 9. **Average annual mean temperature for Zimbabwe downloaded from Climate Change Knowledge Portal (CCKP) (Available at: https://climateknowledgeportal.worldbank.org/country/zimbabwe/climate-data-historical). 

## Data Availability

The data supporting the conclusion of this article are available by the authors without undue reservation. These data regarding malaria incidence were derived from the following resources openly available at: https://www.who.int/data/gho/data/indicators/indicator-details/GHO/malaria-incidence-(per-1-000-population-at-risk). Additionally, data regarding the temperature can be accessed at: https://climateknowledgeportal.worldbank.org/.

## Abbreviations

CCKP: Climate Change Knowledge Portal
IR: Incidence rate
LDCs: Least developed countries
WHO: World Health Organization
